# Implication of epitranscriptomics in trained innate immunity and COVID-19

**DOI:** 10.2217/epi-2021-0104

**Published:** 2021-07-06

**Authors:** Paramasivam Arumugam, Vijayashree Priyadharsini Jayaseelan

**Affiliations:** ^1^Cellular & Molecular Research Centre, Saveetha Dental College & Hospital, Saveetha Institute of Medical & Technical Sciences (SIMATS), Saveetha University, Chennai, India

**Keywords:** COVID-19, epitranscriptomics, m^6^A modification, myeloid cells, trained immunity

The most devastating disease condition of the century experienced by individuals throughout the world is caused by SARS-CoV-2. Recent data indicates a total of 173,674,509 cases globally, with mortality of 3,744,408 cases affecting 213 countries [1]. The COVID-19 pandemic ravaging mankind has reiterated the importance of maintaining good health and a good immune system. The response to infections by the host is elicited either through innate or adaptive immunity. The immune system is typically divided into two types of responses: innate and adaptive. The innate immune system is an evolutionarily conserved mechanism with monocytes, macrophages and dendritic cells that provides an early and effective defense against invading pathogens and other potential threats to the host. Innate immunity was once considered to be a non-specific mechanism with a short span of memory. The adaptive immune system produces a more specialized line of defense, developing specific long-lasting memory, which protects the organism against later encounters with the same pathogen. Numerous pieces of evidence have supported the fact that innate immunity could also sustain immunological memory mimicking an adaptive immune response [2,3]. Interestingly, certain vaccination models developed so far in mammals are known to strengthen immunological memory, contributing to protective effects against reinfection, independent of the adaptive mechanisms. In line with this, it has also been indicated that the fungal cell-wall component β-glucan non-specifically induces enhanced secondary responses in mouse and human cells [2,3]. This form of innate immunity is termed as ‘trained innate immunity’. There is a growing body of evidence that indicates the central role of epigenetic mechanisms in the regulation of trained innate immunity against different types of disease [2,3]. However, the exact mechanism of trained innate immunity regulation is not fully understood. Accumulating data strongly suggests that epitranscriptomic mechanisms play important roles in immune cell activation, innate immune regulation and viral infections [4]. Herein, we set out our vision on how epitranscriptomic modification (RNA epigenetics) regulates trained innate immunity and responses to a variety of immunological diseases.

The molecular mechanisms underpinning the induction, maintenance and regulation of innate immune response depend on the complex interplay between many different metabolic pathways and the epigenetic machinery of the cell. Metabolic reprogramming and the epigenetic modification of innate immune cells serve as major pillars in the induction of trained innate immunity. Myeloid cells (including monocytes, macrophages, neutrophils, and dendritic cells) are central players in innate immunity, which destroys invading pathogens and repairs tissues. Epigenetic regulation in myeloid cells is very important for cell differentiation and activation in response to developmental and environmental cues. Epigenetic regulation involves post-translational modification of chromatin or DNA, and which is coupled to upstream signaling pathways and transcription factors. Trained myeloid cells have been described as cells responsible for nonspecific defense against reinfection independently of adaptive immunity, and enhanced production of proinflammatory cytokines is characteristic of trained myeloid cells. It has been recently evidenced that the epigenetic changes of the individuals infected by SARS-CoV-2 affects the degree of COVID-19 severity [5]. Ultimately, the epigenetic state of innate immune genes and the surrounding genomic neighborhood are determining the strength of the immune transcriptional response.

Recent reports have shown that when a pathogen enters the body, a series of metabolic alterations take place, which subsequently modulates the activity of many enzymes associated with epigenetic remodeling in myeloid cells [3]. The alteration in methylation status and histone acetylation results in increased chromatin accessibility, easier transcription of multiple genes vital for the innate immune response and improved cell function [2,3]. However, it is well established that epigenetic changes play important roles in trained innate immunity, more recent studies demonstrated that epitranscriptomics plays essential roles in innate immune response to viral infection. Hence, an elaborate exploration into this field of epitranscriptomics could unravel a new dimension of treatment modality and preventive strategies that can operate with a greater precision in case of infectious diseases including COVID-19.

Epitranscriptomics is an emerging field dedicated to the studies on RNA modifications. More exploration into this domain will aid us in resolving complex biological problems with more precise solutions. Although around 170 different chemical modifications have been reported to date in RNAs [4], N6-methyladenosine (m^6^A) is found to be the more abundant internal and reversible methylation modification in coding and non-coding RNAs, which control several pathways of gene expression, including splicing, export, translational efficiency, stability and miRNA biogenesis. The m^6^A-mediated RNA modification is regulated by a ‘hub of vital proteins’ commonly referred to as the ‘writers’, ‘erasers’ and ‘readers’ denoting methyltransferases, demethylases and m6A-binding proteins, respectively [6–12]. It has been shown that RNA-type viruses, including SARS-CoV, showed strong associations with RNA modifications. For example, m^6^A and N6, 2′-O-dimethyladenosine (m^6^Am) modifications have been identified to play crucial roles in the viral life cycle. Especially, which can affect the replication of the virus and the host's innate immunity. A recent study has found that the host m6A modification complex interacted with SARS-CoV-2 proteins to modulate SARS-CoV-2 replication [13]. m^6^A epitranscriptome of Kaposi sarcoma-associated herpesvirus (KSHV) was recently mapped. Tan and colleagues demonstrated that m^6^A modification of mRNA mediates diverse cellular functions via examining the viral and cellular m6A epitranscriptomes during KSHV latent and lytic infection. They also showed that KSHV lytic replication induces a dynamic reprogramming of the viral epitranscriptome itself and suggested that KSHV, m^6^A and the reader protein YTHDF2 acts as an antiviral mechanism during viral lytic replication [14].

The m^6^A modification is known to promote immune cells activation, maturation, function and innate immune responses [7,12]. Transcriptome-wide investigation of m^6^A modification has been adequately supported by the advent of high-throughput sequencing technologies in recent years. Novel findings reiterate the potential role of m^6^A modification in enhancing gene expression associated with the regulation of innate immune response [6]. Histone modification is a dynamic process with vital roles in the differentiation and activation of myeloid cells. These key epigenetic marks control chromatin structure and gene expression patterns, thereby impacting on various important cellular phenotypes. The induction of trained immunity in innate immune cells is associated with histone modification, which influences the expression of proinflammatory cytokines and intracellular signaling molecules by attracting transcription factors and other proteins [2,15].

More recent studies have demonstrated that m^6^A methylation and its regulators control histone modifications [12,15–19]. Furthermore, this m^6^A modification destabilizes transcripts encoding histone-modifying enzymes and enhances the expression of proinflammatory cytokines [15,16]. The functions of histone and non-histone proteins are highly regulated by post-transcriptional modification such as lysine methylation. The acquisition of H3K27ac and the consolidation of H3K4me3 are two key epigenetic marks associated with the expression of genes associated with innate immune response in immune cells and trained innate immunity [2]. Despite this evidence, questions arise as to how H3K27ac and H3K4me3 histone marks are associated with active transcription and trained innate immunity. Recent findings have demonstrated that both m^6^A methylation and histone modifications including H3K27ac and H3K4me3 to be essential for cell state transition [15,17,18]. Also, the KDMs are potent modulators of innate immunity, which is controlled by m^6^A regulators in an m^6^A-dependent manner [15,18].

The trained immunity is postulated to be driven by epigenetic marks, transcriptomic tags and functional reprogramming of hematopoietic stem cells of bone marrow or mature macrophages, that largely relies on the CCAAT/C/EBPβ, a potential transcription factor [3]. Studies have also reported that m^6^A modification regulates a network of genes required for human bone marrow mesenchymal stem cells differentiation [6], maturation and differentiation of myeloid cells, as well as adipogenesis by activating C/EBPβ [20]. These findings add onto the existing knowledge on the regulation of gene expression that involves crosstalk between RNA methylation and histone modification associated with innate immunity.

Bacillus Calmette–Guérin (BCG) is a live attenuated vaccine, used in the prophylaxis for tuberculosis. Recent research findings have proposed that BCG vaccine can induce trained immunity and offer protection against multiple viral infections [2,3]. It has been shown that pro-inflammatory cytokines, such as IL-1β, IL-6 and tumor necrosis factor production are enhanced in the BCG-vaccinated individuals. Further epigenetic changes induce innate immune cells to retain the memory which expresses as antiviral response [2,3]. Accumulating evidence has supported the fact that BCG vaccine could induce trained immunity offering a significant protection against respiratory tract infections including viral infections [21]. The rapid dissemination of COVID-19 and its key variants underscores the need for more specific methods of prophylaxis, diagnosis and treatment. Clinical trials initiated by several countries have tested the capacity of BCG against COVID-19 [3,21]. We, therefore, hypothesize that the external stimuli including BCG vaccine can induce trained innate immunity through epitranscriptomics modifications of histones rendering significant protection against viral infections including COVID-19.

Taken together, we set out our vision to probe into mechanisms of targeting innate immune cells and modify trained innate immunity via, epitranscriptome modifications to accomplish long-term and broad-spectrum benefits across a range of immunological disorders. The present editorial review throws light on the multiple means by which m^6^A modification regulates innate immune systems leading to trained immunity. More research into the field of epitranscriptomics related to trained innate immunity is sure to bring in a revolution in the prophylaxis and redesigning new therapeutic methods.
